# Smart Winery: A Real-Time Monitoring System for Structural Health and Ullage in Fino Style Wine Casks

**DOI:** 10.3390/s18030803

**Published:** 2018-03-07

**Authors:** Eduardo Cañete, Jaime Chen, Cristian Martín, Bartolomé Rubio

**Affiliations:** 1 Department of Electronics and Computer Engineering, University of Córdoba, Ctra. Madrid-Cádiz Km. 396, 14071 Córdoba, Spain; 2 Department of Languages and Computer Science, University of Málaga, Boulevar Louis Pasteur 35, 29071 Málaga, Spain; hfc@lcc.uma.es (J.C.); cmf@lcc.uma.es (C.M.); tolo@lcc.uma.es (B.R.)

**Keywords:** sensor, real time, monitoring, distributed, IoT, wine, cask

## Abstract

The rapid development in low-cost sensor and wireless communication technology has made it possible for a large number of devices to coexist and exchange information autonomously. It has been predicted that a substantial number of devices will be able to exchange and provide information about an environment with the goal of improving our lives, under the well-known paradigm of the Internet of Things (IoT). One of the main applications of these kinds of devices is the monitoring of scenarios. In order to improve the current wine elaboration process, this paper presents a real-time monitoring system to supervise the status of wine casks. We have focused on a special kind of white wine, called Fino, principally produced in Andalusia (Southern Spain). The process by which this kind of wind is monitored is completely different from that of red wine, as the casks are not completely full and, due to the fact that they are not renewed very often, are more prone to breakage. A smart cork prototype monitors the structural health, the ullage, and the level of light inside the cask and the room temperature. The advantage of this smart cork is that it allows winemakers to monitor, in real time, the status of each wine cask so that, if an issue is detected (e.g., a crack appears in the cask), they can act immediately to resolve it. Moreover, abnormal parameters or incorrect environmental conditions can be detected in time before the wine loses its desired qualities. The system has been tested in “Bodegas San Acacio,” a winery based in Montemayor, a town in the north of Andalusia. Results show that the use of such a system can provide a solution that tracks the evolution and assesses the suitability of the delicate wine elaboration process in real time, which is especially important for the kind of wine considered in this paper.

## 1. Introduction

The Internet of Things (IoT) [1] has progressed substantially in the last few years thanks to the continuous advances in many areas such as miniaturization and electronic technology. Nowadays, the IoT is a flagship to move the real world into a digital one, and it is present in many areas, from houses to cities [2]. The IoT is intended to optimize the processes in many areas through resource-constrained devices, which provide the capacities to sense and actuate in these areas over the Internet. Nevertheless, it is well known that the underlying devices in the IoT have serious limitations in terms of storage, processing, and battery power. To enable current disruptive paradigms, such as big data, to access and process the amount of data generated from the IoT, including data in real time, the IoT needs to have an upper layer to meet this demand. Cloud computing can supplement (1) the limitations of associated devices in the IoT in terms of storage and computing, and (2) the requirements of complex analysis, scalability, and data access [3]. Cloud computing offers a virtualized infrastructure that provides unlimited resources in terms of storage and processing power [4], the main drawbacks in the IoT. The integration of these two technologies brings to the IoT (1) a necessary layer to address its main limitations and (2) multiple data sources for extracting knowledge to cloud computing [3].

The IoT is a promising technology in areas with high manual involvement, such as agriculture and viticulture, thanks to its capacities to automate processes [5]. Viticulture is an ancient process. According to the earliest archaeological evidence, the first grape wine dates back to 7000 BCE in China [6], and the first wine production dates back to 4100 BCE in Armenia [7]. Since then, wine production has expanded globally, and today wine is one of the most consumed alcoholic drinks in the world [8]. Technological advances in harvest, processing, and elaboration over time have influenced this expansion. However, there is still a lack of automated control in the elaboration of wine, which usually implies high manual involvement [9]. As discussed, the IoT is especially suitable for environments such as viticulture, which require a high amount of human resources. The key idea is to automate human processes and reduce the knowledge obtained manually to an automatic retrieval process, which would reduce actuating times in cases of disturbance. Therefore, the adoption of IoT in this area could optimize current wine production processes.

In the region of Andalusia (Southern Spain), a type of white wine called Fino [10] is produced. In Spain, it is one of Andalusia’s most appreciated wines and is internationally well known as sherry. Fino is different in several ways from other wines. It is created with a type of yeast [11] and is the driest and palest of the various types of Spanish wines. Regarding the production process and compared to other varieties, it is not aged as long and should be drunk as soon as possible once the bottle is opened, as exposure to air can cause the wine to lose its flavor. This type of wine owes its light and fresh flavor to the yeast used in its production.

During the wine aging process, a certain portion of a wine’s liquid volume evaporates and escapes from the cask. This phenomenon, known as “merma” [12], has to be controlled, as it is very important to maintain the level of wine inside of the cask during the aging process. In the case of red wines, the cask has to be completely full, but for Fino wine, ullage (“ullage” refers to (1) the air space between the wine and the top of the cask and (2) the evaporation process that leads to the air space) exists and has to be kept constant during the aging process. If these levels are not controlled, the quality of the wine will suffer, or, even worse, the wine will spoil. There are other critical issues that are hard for the winemaker to control. Casks used to age Fino wine are more prone to breakage due to the fact that they are not renewed very often. Cracks in the cask are not uncommon and can lead to a considerable loss of wine from that cask (they contain about 560 L) before the first evidence of the cask’s deterioration can be detected. In short, this can incur a high economic cost, especially in large scale wineries. For these reasons, we propose using the IoT to instrument the Fino wine casks in order to provide winemakers with a tool to monitor the structural health and important parameters of the casks stored in their wineries in real time.

In this paper, a real-time monitoring system for monitoring the structural health and ullage of Fino wine casks is presented. One of the main components of the system is the smart cork, which is seamlessly integrated in the Fino wine cask and used to obtain information about the room temperature, the humidity, and the ullage within the cask. This information is used to detect possible cracks and to track the level of wine inside the cask with the goal of maintaining the balance between ullage and wine. All this information is uploaded and managed by a cloud platform where final users can set alarms (e.g., receiving a warning when the wine level falls below a certain threshold) and monitor each cask individually. The system has been tested in a real environment, specifically, at a winery called “Bodegas San Acacio” (Bodegas San Acacio: http://bodegasanacacio.com/), which is located in Montemayor, a town in the south of Córdoba. This winery produces around 2,050,000 L of wine per year and its cellar has more than 850 casks. The wine is produced under the “DO Montilla-Moriles” [13]. “DO (https://www.wine-searcher.com/wine-label-eu.lml)” is related to the PDO (Protected Designation of Origin) concept; according to the EU definition, PDO products are “produced, processed and prepared in a given geographical area, using recognized know-how.” Results obtained during the tests show that the use of the system presented in this paper can provide a solution for tracking the evolution and assessing the suitability of the delicate wine elaboration process in real time.

The rest of the paper describes the architecture of the system and studies its feasibility based on a real case study. In Section 2, related work is discussed. The monitoring and application requirements are presented in Section 3. Section 4 describes the system and the IoT platform. In Section 5, results and evaluation of the system are provided. Finally, Section 6 details the conclusions.

## 2. Related Work

A number of sensor-based monitoring systems have been developed over the last few years intended to improve the quality of wine during its elaboration. Most of them are focused on controlling the fermentation process in real time. Fermentation is the process in which a substance breaks down into a simpler substance. Microorganisms such as yeast and bacteria usually play a role in fermentation processes in the creation of beer, wine, bread, kimchi, yogurt, and other foods. However, there are other systems whose goal is to monitor the maceration process or simply the temperature of the wine. Maceration is the process of soaking crushed grapes, seeds, and stems in a wine must to extract color and aroma compounds as well as tannins. This is where red wines get their color and tannins, and it is the lack of maceration that makes white wines light in color and nearly tannin free. In this section, some of the most recent and relevant related work is described. To the best of our knowledge, we are the first to propose a real-time monitoring system based on a smart cork, able to monitor the structural health of the casks, the room temperature, and the level of light and balance between the wine and air inside the casks. This is particularly important in the elaboration process of the kind of white wine called Fino. Last but not least, a cloud computing platform is integrated to facilitate the scalability of the system.

In [14], a low-cost module to monitor the grape fermentation process is presented. The module collects information about temperature, wine acidity (pH), alcohol, and carbon dioxide that is wirelessly sent to a server so that small winemakers can control this crucial process in real time. A similar approach is presented in [15], where the authors present a low-cost sensor able to monitor the fermentation process in real time by controlling the temperature within the tank where the wine is evolving. This information is transmitted to the winemakers through a Bluetooth connection. A different approach to control the fermentation process is presented in [16], where a new sensor is used. The authors designed an aluminum-nitride-based piezoelectric microresonator that acts as a density sensor able to detect the decrease in sugar and the increase in ethanol concentrations during the grape must fermentation process with a high resolution.

Wine-SBS [17] provides a smart cask to monitor the wine fermentation of the Debina variety of semi-sparkling wine. The system, which is installed inside each cask, continuously monitors aspects of the wine such as color, transparency, pH, pressure, temperature, and alcohol levels. The development makes use of WiFi, which considerably increases the power consumption of the IoT device. All measurements are uploaded to a web server, which enables the monitored data to be accessed.

The authors of [18] propose an optoelectronic sensor device for monitoring the maceration of red wine, providing an instrument based on absorbance measurements aiming to not only assess the chromatic characteristics of finished red wines but also supervise the gradual maceration of fermenting grape musts. In [19], the authors propose creating an e-nose (based on resistive metal oxide (MOX) sensors), an e-eye (based on CIELAB coordinates (CIELAB is the second of two systems adopted by the Comission Internationale de l’Eclairage (International Commission on Illumination) (CIE) in 1976 that better showed uniform color spacing in their values)), and an e-tongue (based on voltammetric sensors) to monitor the aging of red wine. However, the data is not accessible beyond the evaluation results.

In [20], the authors present a system to monitor the aging process of tawny port wine. The system adopts an architecture similar to the work presented in this paper, in that sink nodes and an IoT platform are used. However, the industrial-certificated sensors used have a high cost and a wired connection using RS-485 and RS-232C standards. Installing such a system and wiring all of the sensors requires a great amount of human resources, and the space of the winery is reduced. This contrasts with our solution, which is a wireless, plug-and-play solution and does not require any configuration beyond a connection to power and installation in the cask.

A system to monitor the malolactic fermentation (MLF) process is presented in [21]. The MLF consists in bacteria that is used to improve the wine quality and has to be properly monitored to prevent off-flavors. This process allows the pH and the temperature inside the cask to be monitored. However, an insufficient amount of information about the infrastructure and hardware components is provided to be sure, and this solution does not provide integration with any cloud infrastructure. Further, data is visualized through a PC through a text file. In our solution, configurable charts and alerts can be defined.

An open-source and low-cost monitoring system for precision enology is presented in [22], where the authors present a sensor network that is integrated into the silicone bungs of the casks able to monitor enological parameters, such as pH and temperature during the wine aging process.

A low-cost energy-saving bung to monitor red wine casks is presented in [23]. The prototype is embedded within the cask bungs and monitors the temperature and ullage inside the casks. It should be noted that the prototype can run on batteries for 12 months at a cost of 27 Australian dollars for each prototype (not including the cost of sink nodes and central servers). The information is sent to a central server that analyzes the data and sends out alerts when necessary.

In [24], an IoT monitoring system for vineyards and wineries is presented. The system relies on wireless communication using ZigBee, as in our proposal, and GPRS, but the sensors available focus on vineyard monitoring rather than winery monitoring. The prototype includes one test sensor that has been attached to the bung of a cask but only provides information on the temperature inside the cask.

With respect to price, in [15], the authors present a lower-price prototype, but it only monitors temperature with a lower-precision sensor. In [17], the authors do not provide information about the prototype cost. Based on our estimation, the latest prototype costs approximately 300 euros (€) without an enclosure, almost twice the cost of our prototype (€166). In [19], there is not enough information about the system’s costs and the hardware components used. The cost of the system in [22] is very close to that of our prototype (€150), but that system was designed for different purposes. The approach in [20] uses costly industrial-certified sensors. The cost of a single unit can be five times higher than that of our prototype. In [21], although the authors do not provide enough information about the hardware components, some of their industrial-certified sensors are also costly. Lastly, the system described in [23] is much less expensive than our prototype, but their sensors show a lower precision. Therefore, although our prototype is not the most cost-effective solution, it does provide precise results at a medium cost. According to the winemaker of the cellar where we tested our device, the price of the wine contained in a cask is around €2000. The estimated price of a commercial device based on our prototype is lower than the cost of our prototype, which is 8.3% of the price of the wine cask. The winemaker has suggested it may not be necessary to deploy one cork per cask; rather, it would be ideal to install a cork in casks that are starting to show small cracks.

## 3. Monitoring System Requirements

**Figure 1:**
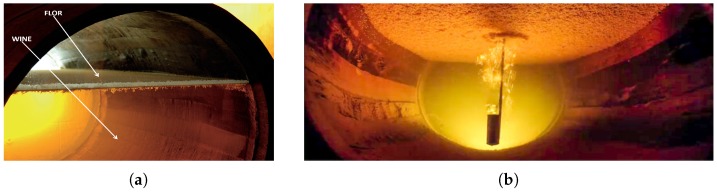
Flor yeast layer. (**a**) Internal view of a Fino wine cask; (**b**) the view of a film of yeasts from the bottom.

From an instrumentation point of view, the system requirements needed to monitor a Fino wine cask are not the same as those used to monitor a red wine cask. This is mainly due to the kind of aging process required to elaborate each type of wine. Red wine uses oxidative aging, which consists of storing and developing wine in wooden butts, where it undergoes slow, physico-chemical development influenced by surrounding conditions. (Oxidative aging is a process used in the elaboration and aging of wines and purposely allows controlled amounts of air to oxidize wine.) In contrast, Fino wine undergoes biological aging [25], a process that takes place under a film of yeasts known as “velo de flor” (see Figure 1), during which the wine develops in a more dynamic way, driven by what goes on within the biological layer formed on its surface by specific, indigenous, ambient yeasts.

**Figure 2:**
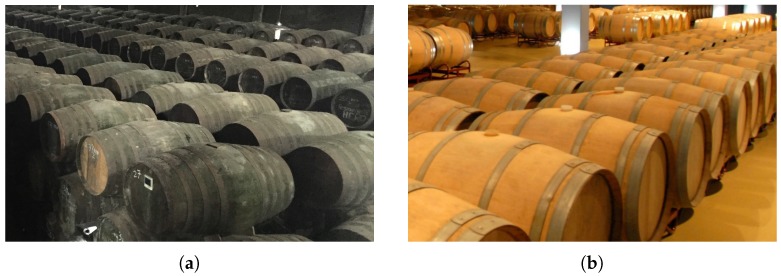
Fino wine cask vs. red wine cask. (**a**) Fino wine cask; (**b**) red wine cask.

The different types of aging are the consequence of having to use completely different types of casks to age each kind of wine. In the following paragraphs, the two main differences between Fino and red wine casks are described:

1. The first difference is that red wine casks used are completely full of wine in order to prevent air from oxidating the wine. In contrast, as shown in Figure 1, in Fino wine casks, there is an air space that is not in direct contact with the wine thanks to the “velo de flor,” which is a protective layer over the top of the wine that shields the wine from over-oxidation.
2. The second difference is that red wine is aged in casks that have to be renewed, at least, every eight years. There are even wineries that change the casks every three years in order to improve the quality of their red wines. On the contrary, the age of a Fino wine cask is not that important. There are casks that are more than 20 years old (see Figure 2), and, as a result, these casks are more prone to breakage.

**Figure 3:**
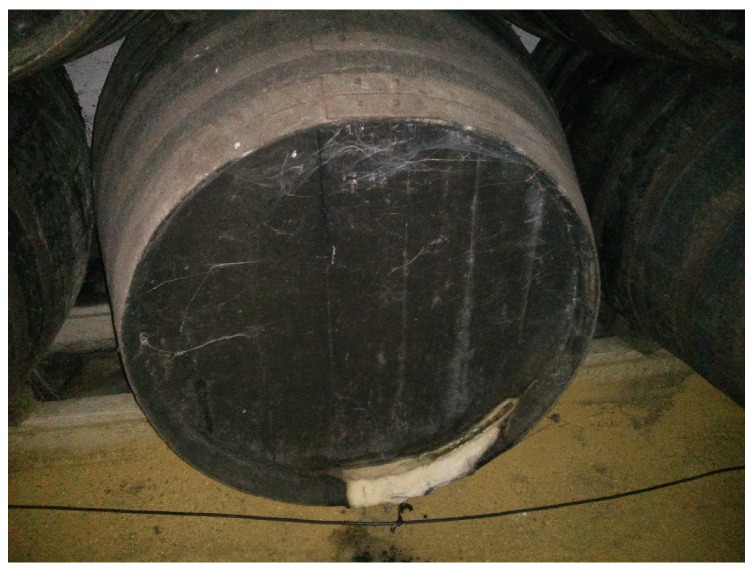
Wine loss due to a crack in a cask.

Based on the special requirements of the wine elaboration process, we identified a set of requirements that our system must meet:

1. The system must not be intrusive. This means that ideally the system must not come into contact with the wine and must not alter the condition of the winery (temperature, humidity, etc.).
2. The system must be easy to install, and non-experts able to replace it. In particular, a wireless and autonomous system is preferable over traditional wired systems. The system must be relatively small compared to the size of the cask and must provide an easy way to check that it is working.
3. The system must be able to monitor and store the following physical parameters: the temperature and humidity of the room, and the level of light and ullage (wine level) within the cask.
4. The system must be able to detect that a crack has appeared in the cask. Figure 3 shows a broken cask from the “Bodegas San Acacio” winery. The issues detected must be notified to the user via the cloud.
5. The information collected by the system must be stored and queried from the Internet in real time preferably through a web application.
6. If an abnormal parameter or issue is detected in the cask the cork must visually notify that something unexpected has been detected (e.g., using a simple LED).

## 4. Winery Monitoring System

**Figure 4:**
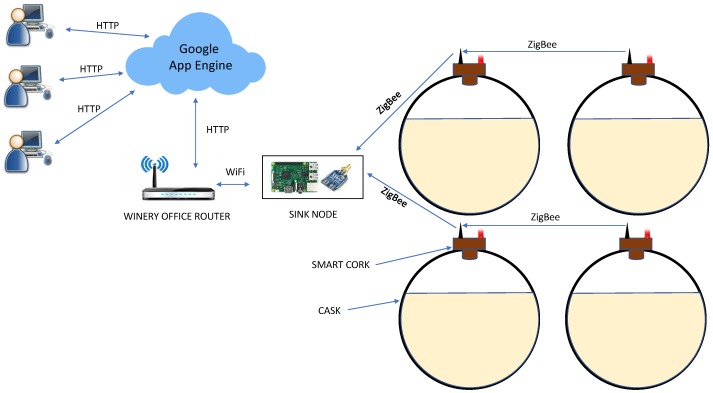
System architecture.

This section describes in detail all the parts of the system used to monitor a winery: the smart cork used to instrument the casks and obtain valuable information from them, its implementation, the communication architecture used to transmit the information from the casks to the cloud, and the cloud platform created to store the data obtained from the casks and provide the end users (e.g., winemakers) with an intuitive, understandable, and flexible way of visualizing this data. Figure 4 depicts the general system architecture deployed in the “Bodegas San Acacio” winery.

### 4.1. Smart Cork Design

**Figure 5:**
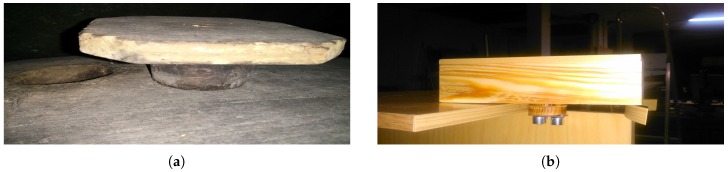
Normal cork vs. the new proposed smart cork. (**a**) Normal Cork; (**b**) smart Cork.

Taking into account the first and second requirements, the cork of the cask is probably one of the best places to integrate the hardware needed to instrument a cask. Figure 5 shows both the traditional cork and the one proposed here. The surface area of the traditional cork has been replaced with a hollow box where the hardware is installed, and the bottom part has been perforated to wire an ultrasonic distance sensor with a microcontroller placed in the hollow side. The proposed cork provides us with a non-invasive monitoring device that offers the following advantages:

1. The hardware is not in contact with the wine.
2. If the monitoring system needs to be fixed, it can be easily removed without affecting the cask and its wine.
3. The price of the new cork (without hardware) and that of the traditional one are similar.
4. Winemakers do not have to change the way they work, which means that the adaptation process is expedient.

### 4.2. Smart Cork Implementation

The prototype was implemented using the Arduino open-source prototyping platform due to its wide community support and the high number of available libraries. The first prototype implemented was tested on a Arduino Mega 2560. In future work, it is expected to use a low-energy variant such as a Moteino in order to extend the lifetime of the system.

**Figure 6:**
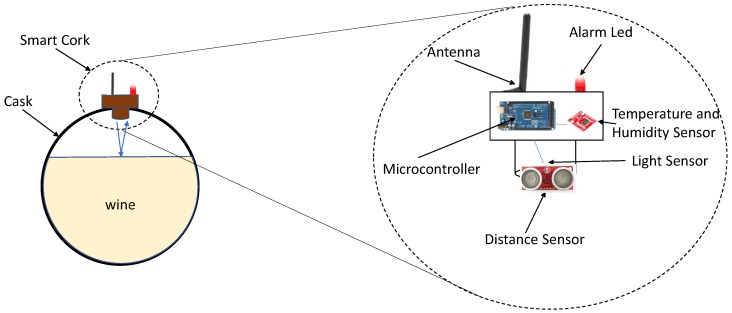
Smart cork hardware architecture.

Figure 6 summarizes the hardware architecture that has been installed in the smart cork. The sensors chosen provide information about important parameters that are essential during the wine elaboration process and whose alteration has a negative effect on the quality of the wine [26]. Table 1 depicts the models and consumption of the different components that have been chosen for the prototype system. The table also shows an approximate cost of the cork prototype components, resulting in a total of €166 per cork. Note that the price is for the prototype and the cost of the wooden enclosure has not been included. A production-integrated solution would significantly reduce the costs. Another communication module would necessarily have to be placed in the sink node. The sink node could be deployed on many more powerful IoT devices such as a Raspberry Pi (54€ approx. price) or a PC.

**Figure 7:**
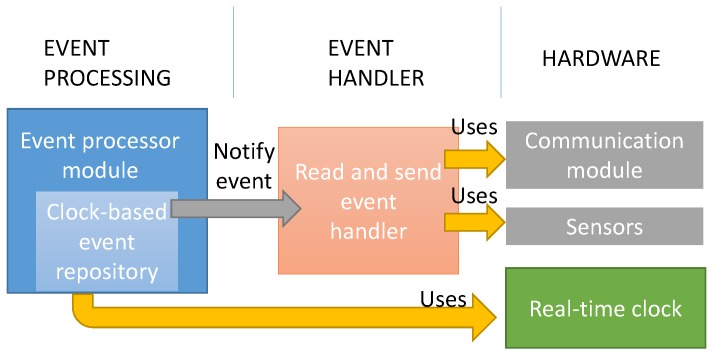
Firmware Architecture.

Figure 7 shows a simple diagram of the firmware architecture. The firmware installed in the Arduino is a simple event-based middleware that carries out actions based on different events that can be defined by the developer. Events are raised by the developer or can be automatically raised by the event-processor module. This module uses a real-time clock module to raise time-programmed alarms. In the prototype program, a total of 144 clock-based events per day have been programmed (one every 10 min). After the event has been triggered by the event-processor module, the firmware carries out the following steps:

1. All the sensors in the board are read together with a timestamp.
2. The data collected from the sensors are stored in an SD card
3. The data are read from the SD card and sent to the sink node.

**Figure 8:**
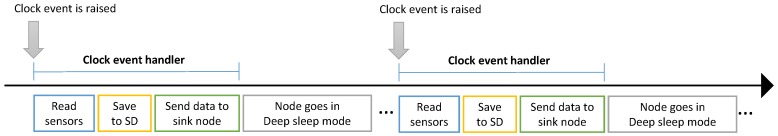
Event schedule of the prototype used in the evaluation.

Figure 8 describes the event schedule used in the tests.

All the components mentioned have been installed in a preliminary wooden case (shown in Figure 5b) that is used as a cork for each of the casks in the winery. The complete system can be powered using two different methods. The first is to connect the board to an electrical plug using a power adapter. The second is to use a chargeable battery. In our tests, we have used a conventional 5 V power adapter and a 2200 mAh 7.4 V Lithium polymer battery.

In order to save energy, the event-processor module checks the time of the next clock-based event to assess how long the board should sleep before the next event is raised. The module then puts the board into deep sleep mode until the next clock-based event. The sleep functionality is provided by the JeeLib (https://jeelabs.org/pub/docs/jeelib/) Arduino library. Communication is turned off when it is not needed (which is most of the time) by means of a sleep pin provided by the module, enabling a long lifecycle of the prototype.

### 4.3. Hardware Components

As stated, the prototype monitors the humidity, temperature, light, and distance from the casks. The Arduino platform was chosen as the hardware-development platform to implement the middleware and the low-level interaction with the hardware components. The Arduino platform is an open source and hardware project that is present in a large number of IoT projects due to its reduced price, its large community, and its easy programming. Moreover, most hardware components such as sensors and actuators provide support for the Arduino platform. Specifically, the Arduino Mega 2560 was chosen among the available boards, since it provides three serial-interfaces and allows the connection of multiple components at the same time such as a debugging port and a wireless communication module.

Mega Sensor Shield V2.4 is a expansion shield for Arduino Mega that provides three Xbee slots to connect communication modules, a prototyping area, and a microSD slot. This expansion shield eased the connection of the wireless communication module and the hardware components, in addition to enabling a microSD slot that is used by the middleware to store all measured information. ZigBee (ZigBee Alliance: http://www.zigbee.org/) was chosen as the communication means for the communication between the corks and the sink nodes. ZigBee is based in the standard for low-rate wireless personal area networks (LR-WPANs) IEEE 802.15.4. IEEE 802.15.4 focuses on low costs and low speeds, and usually also provides support for long-distance communications. It can be contrasted with Wi-Fi, which requires more power but offers more bandwidth [27]. Power is very limited in IoT devices, since most of them are battery-powered. Other communication means, such as Bluetooth, unlike ZigBee, require pairing before they will work and are not designed to enable devices to sleep like our prototype does, which is required in low-power IoT devices and long-term communications.

To obtain the exact time at which sensor data is obtained in the corks and not in the sink nodes, the DS1307 serial real-time clock with a button cell was used. DS1307 is a low-power component that allows the current time to be defined and fetched even when the node has been disconnected. This component is required since Arduino Mega does not provide that functionality. The SR08 sensor was used to obtain the distance from the Fino and the luminosity inside the cask. SR08 is a high perforxmance ultrasonic sensor with a wide range (from 3 to 6 m) that is equipped with an LDR to measure light levels. Finally, the temperature and humidity are obtained with the highly accurate SHT15 sensor with a resolution of 0.01 ${}^{\circ }$C and 0.05% in humidity.

### 4.4. Communication Architecture

The communication architecture (see Figure 4) has been structured in two levels. In the lowest level, there is a powerful node acting like a gateway (sink node) that collects all the information generated by the smart corks (end-nodes) through the ZigBee protocol working in the 2.4 GHz band. In a large winery (which is not our case), where distances are greater, adapting this communication level so that all the casks are able to communicate with the gateway may be necessary. The ZigBee protocol allows multihop routing, which can be used in order to extend the wireless range of the network. Finally, in the highest level, the gateway is connected through WiFi to a WiFi router in the winery’s office, and the information received is sent from smart corks to the cloud using HTTP.

The gateway prototype has been deployed in the system on a chip (SoC) Raspberry Pi 3 model B, which is permanently connected to an electrical energy supply. The Raspberry uses an Xbee S2B ZigBee communication module configured as a coordinator in order to collect the information sent by each of the smart corks in the winery. Raspberry Pi 3 model B incorporates a built-in WiFi, so it is not necessary to include external USB sticks as in previous versions. The gateway, like the smart cork firmware, is executed when devices are started. Therefore, in the case of an interruption such as an power outage, the system returns to its usual behavior when conditions return to normal without the need for human intervention. Moreover, the gateways are previously configured with cloud credentials enabling a plug-and-play (PnP) architecture, which only requires configuration of the WiFi credentials.

### 4.5. Cloud Platform

The data generated by the smart corks are received and managed by the Google App Engine (GAE) [28]. GAE is a platform as a service (PaaS) that facilitates application development for developers, abstracting the hardware, scalability, and high availability requirements of their applications. Application developers only need to focus on what they do best, the application logic, whereas aspects such as an unexpected application growth and a peak demand are dynamically managed by the GAE, thereby avoiding service disruptions and offering high availability. Nowadays, the GAE is widely used for application development and has been adopted by large companies such as Rovio Entertainment [29]. In the context of the work here, the GAE opens the door to the integration of multiple smart corks, even a worldwide adoption, without having to take into account the underlying hardware or to change the application logic.

To receive and make the wine cask information available through the Internet, a representational state transfer (REST) application programming interface (API) has been developed in the GAE. The REST API offers the mechanisms necessary to connect the smart corks with the platform and allow external parties to access the wine cask information in an widely open and standardized way. A web user interface (UI) uses the REST API to allow the application’s users to visualize and manage the cask information. However, the REST API can also be integrated into another system such as the IT infrastructure of the winery owners. The information of each interested party is managed through a permission-based system. The system provides three different access roles: administrator, registered user, and smart cork. The administrator role has access and can manage all functionalities offered by the system, such as creating and updating users and casks and defining alerts based on cork data. The registered users will be comprised of the winery employers, who will be able to visualize the casks and charts with the smart cork sensors’ data. Lastly, the smart corks will be part of the smart cork role that will enable the uploading of the sensor measurements to the GAE.

**Figure 9:**
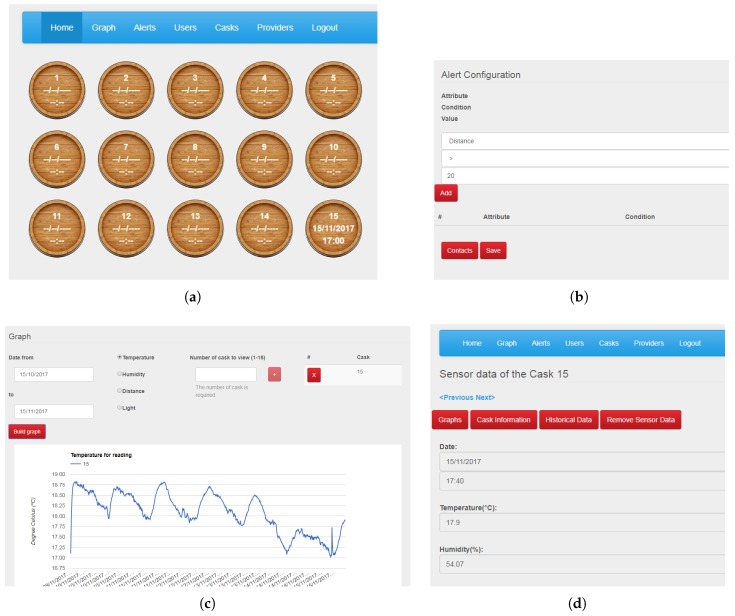
Screenshots of the web UI. (**a**) Overview of the winery casks. (**b**) Alert configuration. (**c**) Graph generation. (**d**) Cask information.

Once a registered user has accessed the system using his/her credentials, the web UI displays an overview of the casks, as shown in Figure 9a. By selecting a certain cask, a form, as shown in Figure 9d, will be displayed where the latest information about that cask can be seen. Furthermore, admin users can also generate graphs of the physical phenomena measured, visualize the cask information, see the historical data, or even remove it. Winery users can only visualize information about the casks and generate graphs. Graphs can also be generated with the information from various casks with the Graphs tab, as shown in Figure 9c. This functionality enables the evolution of different casks to be compared. However, one of the main desired functionalities of the system is the anomaly detection. Knowing about a cask anomaly as soon as possible can reduce its effects. Alerts can be configured though rules in the Alerts tab as shown in Figure 9b. In this case, it is configured to generate an alert when the ullage is greater than 20 cm, i.e., a possible cask leak. Alerts can be associated with contacts, who will receive an e-mail with each alert generated in the system. With the rest of the tabs, admin users can manage the registered users and providers, and the information related to the casks.

To store all this information, the Google Cloud Datastore (GCD) system was used. The GCD is a NoSQL [30] document database designed for high performance, automatic scaling, and ease of application development since it does not require initial configuration. The result is that tables are dynamically created without human intervention. The datastore is designed for querying large datasets, so latency is reduced when the substantial amount of data generated by the IoT is accessed. However, this datastore also has limitations with respect to traditional SQL-relational databases, as there is no support for multiple properties, joining operations, or subqueries. However, these are not necessary in the context of this project.

## 5. Evaluation and Results

Once we had built the smart cork comprised of temperature, light, humidity, and distance sensors and developed the software to manage the information obtained by these sensors, we then tested the whole system under real-world winemaking conditions. Sensors used in this prototype are high-precision sensors and do not require any user calibration. In this case, sensors measurements were tested with other sensors at our laboratory before deployment in the winery. In addition, the change in ullage was confirmed with the winemakers who informed us that they had to add wine to the cask to keep the ullage constant.

**Figure 10:**
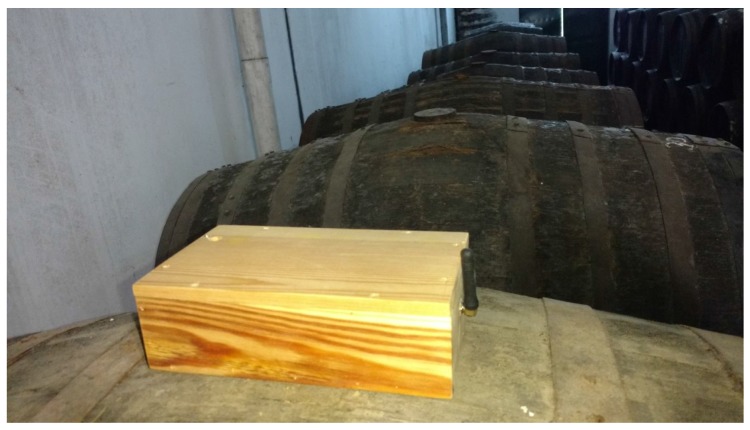
Smart cork installed in a winery based in Montemayor (Córdoba).

A smart cork was installed in a cask located in Montemayor, in the province of Córdoba (see Figure 10), to monitor, on the one hand, the ullage and the level of light inside the cask and, on the other hand, the temperature and humidity of the room. The monitoring process in our test was carried out for 19 days, and the sampling rate was set to once every 10 minutes for all the sensors.

**Figure 11:**
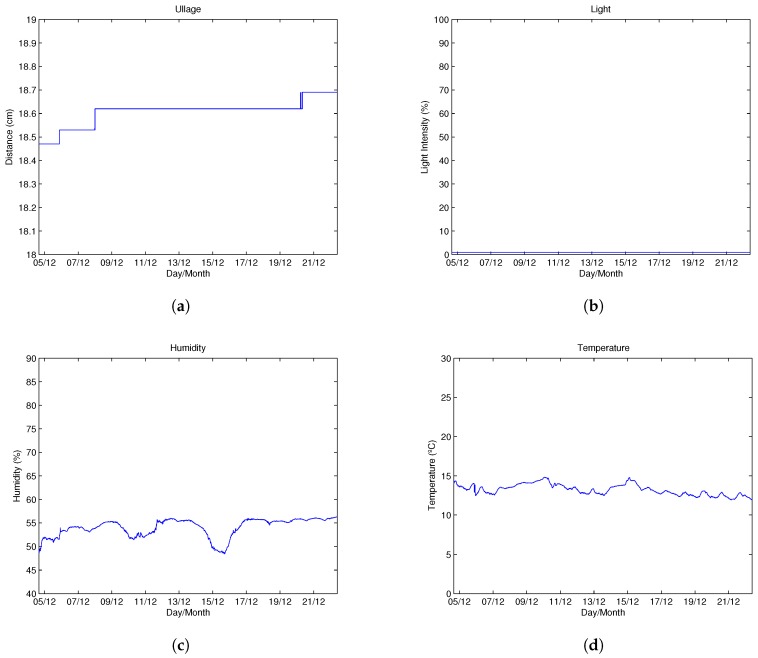
Wine cellar sensor readings. (**a**) Ullage (cm). (**b**) Light intensity (%). (**c**) Humidity (%). (**d**) Room temperature (${}^{\circ }$C).

All the readings obtained by the smart cork through its sensors are visualized in Figure 11 (a-ullage, b-light, c-humidity, d-temperature). Figure 11a shows how ullage slightly increases as time passes. For example, on 9 December, the ullage increased from 18.46 to 18.62 cm and from 18.62 to 18.69 cm on 20 December. These small increases are very likely due to slow evaporation losses, as expected. A large increase in the ullage would have been caused by a crack in the cask, but it would have been difficult to test this in this deployment, as it is something that might occur after 4 days or even after 6 months. Currently, we are working on a better prototype that it is to be deployed and tested over a longer period of time (6–12 months).

**Figure 12:**
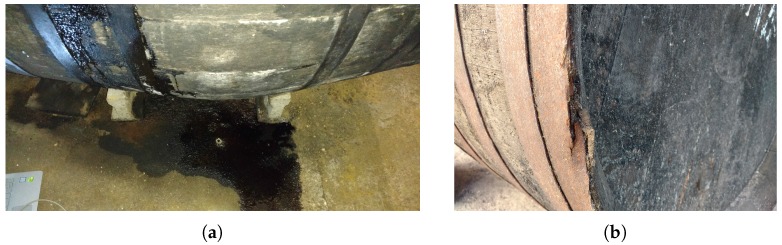
Defective casks. (**a**) Cask losing wine through small cracks. (**b**) Defective cask due to a received blow.

Figure 11b shows that the interior of the instrumented cask was completely dark for the 19 days that the system was gathering information. As we explained, a signal of light indicates that a crack has appeared in the cask and, as a result, a considerable loss of wine, but the system indicates that neither of these two events occurred. After this experiment, according to the “Bodegas San Acacio” winemaker information, two new defective casks were detected, shown in Figure 12:

1. One cask was emptied by the operators as it was losing a large amount of wine due to a blow received by a machine (Figure 12a).
2. There was another cask installed in the cellar that lost wine slowly but continuously (Figure 12b).

We placed the smart cork in both casks to detect light inside the cask. While the light sensor was reading data with a frequency of 0.5 ms, we passed a powerful flashlight through the zones where wine was leaking, and we realized that the sensor was not able to detect any change in the variation in luminosity inside the cask. Therefore, we reached the conclusion that the structure of the small cracks that appear in the cask do not allow light to pass inward but nevertheless allow the wine to filter through them.

Figure 11c reveals that the humidity falls always inside the range 45–60%. The winemaker tries to maintain the level of humidity constant by irrigating the cellar of the winery which has a special layer of sand called ‘albero’ that helps to maintain the level of humidity for a longer period of time. In spite of this, the sensor shows that the humidity was far from reaching the proper level, which is around 80%.

Finally, Figure 11d shows the room temperature of the winery. From a wine-maturation-process point of view, the most important thing is to ensure that the temperature of the room where the casks are stored is within the range 10–15 ${}^{\circ }$C. The temperature data confirms that the winery temperature is within the normal range and that the temperature levels remain relatively constant throughout the day, regardless of outdoor temperature.

## 6. Conclusions and Future Work

To the best of our knowledge, we have developed the first smart cork prototype designed to monitor casks with Fino wine. These casks are particular because there is high ullage and they are not renewed very often. In this work, we focused on Fino wine casks, which, unlike red wine casks, are not completely full and are used for many more years, making the monitoring process different. The process by which Oloroso, Amontillado, and Pedro Ximenez are aged is similar to that of Fino, so our smart cork could be used with these other wines as well. The test carried out over 19 days in a real environment, specifically in “Bodegas San Acacio,” shows that the smart cork is able to monitor the structural health of the cask, including the ullage of the cask, the room temperature, and the humidity; however, our tests show that small cracks that allow the wine to filter through them do not produce a significant light intensity variation. The system can provide a solution for tracking the evolution and assessing the suitability of the delicate wine elaboration process in real time, which is especially important for the kind of wine considered in this paper. Furthermore, the smart cork has been integrated within a complete system through which information is not only obtained from the casks but also sent to the cloud, stored, and graphically visualized for winemakers. In future work, we wish to improve many aspects of this prototype: reduce its size, reduce its energy consumption, create a new enclosure with a 3D printer, add new sensors (pH, alcohol, etc.), and add prediction algorithms that notify winemakers when the cask has to be filled.

## Figures and Tables

**Table 1:** Hardware components.

Component	Type	Protocol	Consumption	Approx. Price	Use
SHT15	Humidity and temperature	Digital two-wire interface	4 × $10^{-7}$/1.8 × $10^{-7}$ mA (standby, average)	€35	Measure temperature and humidity room
SRF08	Distance and luminosity	I2C	3/15 mA (standby, average)	€42	Measure ullage and structural health of the cask
DS1307	Real-time clock	I2C	1.5/0.2 mA (ACTIVE/ STANDBY)	€12	Measure time and issue periodical events (such as periodical reading of sensors)
Xbee S2B	ZigBee communication	Serial port	15/47/117 mA (IDLE/RX/TX)	€24	Send information to the sink node
Mega Sensor Shield V2.4	Shield extension for Arduino: Xbee sockets, SD card, and sensor pins	SPI/I2C/ Serial	20/13 mA (ACTIVE/STANDBY)	€18	SD card, sensor communication interface
Arduino Mega 2560	Hardware development platform	SPI/I2C/ Serial	42/21.5 mA (ACTIVE/STANDBY)	€35	Smart cork sensor management and data acquisition and communication
